# Minority report: can minor parents refuse treatment for their child?

**DOI:** 10.1136/medethics-2019-105702

**Published:** 2020-02-14

**Authors:** Helen Lynne Turnham, Ariella Binik, Dominic Wilkinson

**Affiliations:** 1 Department is Paediatric Critical Care, Oxford University Hospitals NHS Foundation Trust, Oxford, UK; 2 Philosophy, McMaster University, Hamilton, Ontario, Canada; 3 Oxford Uehiro Centre for Practical Ethics, University of Oxford, Oxford, UK

**Keywords:** children, competence/incompetence, decision-making, minors/Parental Consent, newborns and Minors

## Abstract

Infants are unable to make their own decisions or express their own wishes about medical procedures and treatments. They rely on surrogates to make decisions for them. Who should be the decision-maker when an infant’s biological parents are also minors? In this paper, we analyse a case in which the biological mother is a child. The central questions raised by the case are whether minor parents should make medical decisions on behalf of an infant, and if so, what are the limits to this decision-making authority? In particular, can they refuse treatment that might be considered best for the infant? We examine different ethical arguments to underpin parental decision-making authority; we argue that provided that minor parents are capable of fulfilling their parental duties, they should have a right to make medical decisions for their infant. We then examine the ethical limits to minor parents’ decision-making authority for their children. We argue that the restricted authority that teenagers are granted to make medical decisions for themselves looks very similar to the restricted autonomy of all parents. That is, they are permitted to make choices, but not harmful choices. Like all parents, minor parents must not abuse or neglect their children and must also promote their welfare. They have a moral right to make medical decisions for their infants within the same ‘zone of parental discretion’ that applies to adult parents. We conclude that adult and minor parents should have comparable decision-making authority for their infants.

## Case scenario

A 15-year-old girl, M, has a long-standing needle phobia. M falls pregnant, and during the delivery of her term baby, B, refuses to have an epidural anaesthetic and intravenous cannula. This refusal is respected by M’s doctors, and the delivery is uncomplicated.

M has been supported during her pregnancy by a teenage parent support group. She is assessed to be capable of caring for B, and B is discharged into M’s care on the second day of life. However, on day 7, the baby develops apnoeas and is brought to hospital by M and her own mother, G, with whom they live. There are signs that B has sepsis. As part of his investigation and treatment, the paediatricians plan to insert an intravenous cannula and perform a spinal tap (lumbar puncture, LP) to exclude meningitis.

M consents to an intravenous cannula for B and administration of antibiotics. However, she finds the process so distressing she refuses consent for B to undergo the LP.

B’s grandmother supports the medical plan for an LP for B. The child’s father, F, who has recently turned 18, is not registered on the birth certificate, and has not shown an interest in him.

Optimal medical management for B is an LP. This is uncomfortable, but low risk. Without the LP, he will require a prolonged course of intravenous antibiotics in hospital (at least 2 weeks). If the procedure confirms that B does not have meningitis, he will likely be able to cease his intravenous antibiotics and be discharged home after 3–5 days. There are also implications for long-term follow-up.

Should the medical team proceed with the LP against M’s wishes (including seeking a court order, if required)? If G or father give permission, would that suffice?^1^

## Introduction

Rates of teenage pregnancy are falling in Western societies,^1^ but there remain an important number of children born to parents who are themselves not yet adults (in this paper, we will refer to these as ‘minor parents’)^2^. In the UK, there are approximately 18 conceptions each year per 1000 female minors under the age of 18 and 3 per 1000 in minors less than 16.^1^ ^3^ Mothers who are minors are more likely to have babies who are premature and or have low birth weight; consequently, their offsprings have a greater risk of medical problems during childhood.

In many such situations, ethical questions do not arise, since minor parents, just as adult parents, are strongly motivated to seek medical attention for their child and accept medical advice. However, in some cases, as in the example above, conflict may arise.

There are two questions. First, who should be the relevant decision-maker for the infant? Second, if it is the biological parents, what are the limits on minors’ decision making as parents? Parents are constrained by moral and legal rules protecting the interests of children. But things become more complicated with older minors, who may be regarded as having developing, rather than fully developed autonomous capacities. What are the implications for this case? Does the fact that M is a minor change the nature or limits of her moral authority? Should she have the same rights and leeway as an adult parent in this instance? Our focus in this paper is on the ethical approach to such questions.^4^ We will largely set aside legal questions, acknowledging that the legal approach differs in different parts of the world. We will begin by summarising some of the arguments in favour of parental rights to make medical decisions for their children.^5^

## Who should decide?

An infant’s first parents are the biological parents who conceived him or her. Others might acquire parental rights, formally through adoption or court order and informally if the parent freely gives permission for another person to share those duties and that person freely undertakes them.^2^ In this case, Infant B has a number of persons who might claim parental rights: his mother who is a minor, his father, F, who has now reached the age of legal majority, and his grandmother, G who is assisting M in her endeavours as a parent.

We are focused on the moral question underpinning the claims to parent B. Three compelling arguments favour permitting M to make decisions for B. An argument based on the duties of parenthood, an argument drawing on the shared consequences of decisions, and a parental-interest justification of child-rearing rights drawing on the value of parenthood.

### Duties of parenthood

Parents have duties and obligations towards their children including a duty to promote the interests of the child. These duties are valuable for children, since children are necessarily dependent on adults/parents for their nurturing and protection. But fulfilling this function requires that parents have certain powers and freedoms to make decisions for their children.^3^ The ability to consent to or refuse medical interventions forms one of these powers.

In some situations, minor parents are not capable of undertaking the duties of parenthood. After birth, their infants may be placed in the care of other family members or foster carers. However, in cases where a minor parent freely undertakes and successfully delivers the duties of parenthood, it arguably follows that they should have the same ability, as an adult parent, to make medical decisions.

In the case above, M was judged capable of making medical decisions for herself during her pregnancy and delivery. She was also assessed to be capable to caring for B, and there is no suggestion than B’s illness results from a lack of attention or care.

Biological fathers may also uphold duties to their children, and have a right to make medical decisions. In this case, however, the biological father (F) has not yet played any parental role in the child’s life. If this interruption to his parenthood was through his own free choice, he has neglected his duties and obligations and consequently forfeited his moral right to make this medical decision.^2^ ^6^

M became pregnant as a minor while in the care of her own mother. The grandmother, G, has the duties and obligations of a parent towards her daughter M and as a consequence has some rights regarding M. Does this extend to decision making for her grandson?

**Figure 1:**
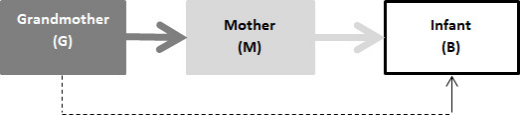
Diagram of duties.

G might have a moral duty to provide emotional, financial and practical help to her daughter, perhaps extending to assist her daughter in making medical decisions. But it is not clear that this requires her to make medical decisions for B or permits her to overrule M. The duty of G towards B is indirect, whereas the duty of M to B is direct (figure 1). Moreover, G’s decision-making rights in relation to M’s own medical care are attenuated because of M’s maturity.

In many circumstances, minor parents may desire the support of their own parents in decision making. If M were to ask her mother to assist with decision making, the grandmother would be justified in participating in the discussions and helping her daughter to understand the risks and benefits of the LP procedure. Additionally, it might be reasonable for the medical team to appeal to G to assist her daughter in making a decision. However, this does not amount to a right to overrule M’s refusal.^7^

Taken together, these considerations suggest that it is M, rather than F or G, who is the morally appropriate decision-maker for B. While parental duties can be relinquished, there is no persuasive reason to think that M has forfeited them.

### Shared consequences of decisions

Parents share a portion of the consequences and burdens of medical decisions made for their children. These might be practical, such as additional need to provide care or limitations in their life choices, financial or emotional (eg, grief or distress at the suffering or death of their child). It would seem, prima facie, fair for those who bear the consequence of decisions to at least have some say in those decisions.

For a minor parent who is the primary carer of a child, the consequences of decisions bear significantly on her and, as with adult parents, it seems reasonable that she makes medical decisions for her child. However, other family members (eg, grandparents) may have similar claims to be involved in decision making.

In the case, M currently lives with and is supported by G. This may mean that G will share in the burdens of decisions (eg, if baby B has a prolonged hospital stay, the need to come back and forth to attend the hospital). It is possible that this living arrangement will continue, and in some situations (where G will be a primary carer for a long period, or bear the financial costs of treatment) that might give her a moral claim to make medical decisions. However, in many cases, including in this example, it is likely that over time the mother and child will come to live independently of grandparents. Where that applies, it does not seem to give G a strong claim to be the primary medical decision-maker for B. If M were a young adult, still living with her parents, we would not usually think that the grandparents had a right to make medical decisions for the grandchild.

### Value of parenthood

Another line of argument proposes that the right to parent is fundamental and that parenting offers a unique good that cannot be achieved in other ways. Brighouse and Swift argue^4^ that relationships between parents and children have a ‘different moral quality, make a different kind of contribution to their flourishing, and so are not interchangeable with other relationships’.^4^ Given that this relationship is irreplaceable, it should be protected.^4 5^

Gheaus builds on this view, arguing that adequate parents have the moral right not only to parent but to parent their own biological babies by drawing on empirical realities of pregnancy.^6^ The infant in this case is only 7 days old but M has already experienced significant physical, psychological, social and financial costs and may have developed an intimate bond with the baby.^6^ These features of pregnancy, combined with the value of parenthood forms the basis for the prima facie moral right for a parent to raise her biological child.

To sum up, the ethical reasons to grant parents a right to make medical decisions for their children appear to apply to minor parents just as they do for adult parents. One difference between minor parents and adult parents is that the duties and burdens of parenthood may be partly shared (particularly with grandparents). This may give grand parents some ethical claim to have a say in medical decisions, (though arguably this would also apply to some adult parents, where grandparents are heavily involved in the parenting role). However, in most situations, the role of grandparents will be indirect—supporting decisions, rather than making decisions.

Yet, while the shared consequences of decisions might give parents some claim to make decisions, it is the child who will bear the greatest burdens of medical decisions made for him or her. This is one important reason for limits being applied to decisions that any parent can make for their child. Should the limits of parental autonomy differ for a minor parent?

## What are the limits to decisions for a minor parent?

If minor parents, like M, are permitted to make medical decisions for their children, should they have the same range of decisions as an adult parent? To assess this question, it will be useful to first assess the range of decisions adult parents can make for their children. We will compare this with the decisions that mature minors are able to make for themselves.

### The limits for decisions of adult parents

While parents are usually thought to have a right to make decisions for their young children, no parent can make unlimited decisions for their infants; parents must provide children with basic goods that are necessary for flourishing,^7^ and they must not abuse or neglect them.

The ‘best interests’ of the child are widely accepted as a fundamental ethical principle for decisions about children.^8 9^ However, health professionals do not over-rule parents whenever they appear to be making suboptimal decisions. Instead, where there is uncertainty of outcome or where there is more than one medically reasonable alternative, the choices of parents are respected, unless the decision for their child poses a significant risk of serious harm.^10^ Society is morally, and also legally, justified to interfere with the parenthood of parents who harm their children.

In paediatric practice, the range of decisions that parents are ethically entitled to make for their children has been dubbed the ‘zone of parental discretion’. Gillam describes this as ‘a morally legitimate space’ between decisions that are optimal for that child, and decisions that are harmful to the child.^11^

A paradigm example of a suboptimal decision that adult parents are permitted to make is refusal of routine immunisation. It is accepted in most societies that parents can choose to have their child vaccinated or not: the decision to vaccinate falls within the zone of parental discretion. There are a range of other suboptimal choices that also fall within the zone of parental discretion.^12^ ^8^

### The limits for decisions of mature minors

Should the limits on parental decision making be different for minor parents? If minor parents are granted decision-making authority for their infants, it potentially follows that the limits of minor parent’s parental autonomy should be constrained in the same way as for adult parents. But this question is complicated because of significant differences between the ways in which the autonomy of adults and minors are respected.

When adults are asked to make medical decisions for themselves, the principle of respect for personal autonomy is paramount. They can make medical decisions that are unwise or even harmful (at least in terms of refusing treatment; they cannot demand medical treatment). Adults are presumed to have capacity; they must be proven to lack capacity before their decisions are justly overruled. A subgroup of adults, without capacity, will have decisions made on their behalf by surrogates.

By contrast, children are assumed to lack capacity. Most decisions are made for them by others, although, a subgroup of minors appear to have the maturity to potentially make medical decisions for themselves. The ethical basis for such restrictions is principally that respect for developing autonomy of the minor should not extend to decisions that would risk significant harm. This difference is reflected in the legal positions for adults and children. In England and Wales, for example, minors may be deemed to be ‘Gillick competent’.^13^ However, where this applies, their choices are more restricted than adults whose capacity is assessed according to the terms of the.^14^ Minors who are deemed competent by the criteria of the Gillick ruling are permitted to consent to medical treatment but there are more stringent tests of their understanding than adults would face—for example, the health profession must be satisfied that they understand ‘all the risks’. Second, in many jurisdictions they have limited rights to refuse treatment.^15^ ^9^

### The limits for decisions of minor parents

Which of these frameworks (if either) should constrain a minor parent’s decision making for an infant? That is, should a minor parent be permitted to make the same set of decisions as an adult parent (ie, within the zone of parental discretion) or should she be subject to a more restrictive framework because of limitations on the decisions minors are permitted to make for themselves?

One reason for holding a ‘restrictive’ view, is that, as noted above minors are not usually given the same rights as adults to make decisions about their own health. If it is justified (for paternalistic reasons) to limit mature minors’ medical decision making about themselves, this would presumably apply even more so to their decisions for their infants. A minor might be regarded as mature (ie, ‘Gillick competent’) to make medical decisions on behalf of her infant. Would she be permitted to refuse necessary medical treatments for her infant? She would not be permitted to refuse necessary medical treatments for herself.

We would argue that it is unnecessary to have a separate approach to the limits of decisions for minor parents. That is because the two frameworks are coextensive: the zone of parental discretion and the constrained autonomy of mature minors share the same ethical boundaries. One plausible way of justifying the limits that apply to decisions for mature minors in their own health is on the basis of the harm threshold. They are deemed to be able to consent to medical treatment without parental input, partly because health professionals should not be offering treatment that would pose a significant risk of serious harm. They are not usually allowed to refuse treatment where such refusal would be harmful.^10^

If this is right, it appears that the zone of parental discretion that applies to adult parents should also apply to minor parents. If, in a case like the one we have described, adult parents would be allowed to refuse an LP, it would be reasonable to allow M to refuse an LP for her infant. On the other hand, if adult parents would be over-ruled, the same should apply to M.

### Objections

#### Variable capacity

One difference between minor parents and adult parents is that there is a reason to be more stringent in assessing capacity in minors. Children have developing intellectual, emotional and practical skills to make decisions. Decisions are also situation dependent, thus children may have capacity to make some decisions, but not others.

In the case of M, her reluctance to consent to the LP because of her fear of needles might be considered to be some evidence of immaturity. Parents must make difficult and unpleasant decisions on their children’s behalf that are in their child’s interests. (We might consider the decision differently if A had refused the LP because she was concerned about the (very) small risk of nerve injury associated with the procedure.) If M’s decisions to decline an LP were irrational, that might lead some to conclude that she lacks capacity to make this decision, even if she has the capacity to make other decisions about her baby’s care.

However, this conclusion would seem to apply double standards to minor parents. For example, adult parents sometimes decline immunisation for their child based on beliefs that have no factual basis or have been discredited.^11^ Their decision might be considered to be irrational, but so long as the decision is not harmful to the child it is nevertheless respected. It seems unfair to regard M’s decision as non-capacitous, when the same decision in an adult parent would not be over-ruled.

#### More serious cases

Some may accept that M is entitled to decline an LP for B, perhaps because the consequences for the baby are relatively small. But they may believe that in other, more serious, cases, her decision should be over-ruled, though the decision of an adult parent would be respected.

Consider a variation of the case. Imagine that B’s birth has been complicated. There was fetal distress, a delay in delivery and B sustained severe hypoxic brain damage. The medical team suspect that B is unlikely to survive and if she survives will be profoundly disabled. They have counselled M that it would be in B’s best interests to withdraw life sustaining therapies and allow him to die but M does not agree.

If M insists on continuing life-sustaining treatment, against the advice of the medical team, would we allow her to make this decision? In most situations, for infants with severe hypoxic brain injury, the decision about continuation or withdrawal of treatment would fall within the zone of parental discretion.^16^ That is on the basis of the medical and ethical uncertainty about B’s outcome and treatment, and the overlap between the interests of the infant and parents.^17^

Some may feel that given the complexity and gravity of the decision, M should not be expected to make this decision. After all, it could be difficult for her to understand the burden of B’s long-term care needs if he survives. It may be extremely challenging for her to imagine B’s (and her own) future life, and to weigh up the ethical considerations in the decision about life-support.

However, this appears to apply equally to many adult parents in this situation. Adult parents may request continuation of treatment, having failed to comprehend (or believe) the infant’s prognosis and having failed to weigh up the relevant ethical considerations. In such circumstances, however, adult parents are not usually over-ruled. If the decision falls within the zone of discretion, their decisions are respected.

It is not clear why this should be any different for minor parents. It is possible that a parent is so overwhelmed by a child’s illness that they are unable to make a decision about treatment. This might apply to both minor and adult parents. Parents need time and support to come to a decision. This, too, would apply to parents of different ages. It may also be that the child’s condition is so serious that treatment should not continue even if the parents wish this. Yet, that implies that the decision is outside the zone of parental discretion, and should apply to both adult and minor parents. Given that M is otherwise capable of parenting and given that adult parents are allowed to make this decision, it appears that M should also be allowed to make this complex decision.

## Conclusion

We have focused on an ethical analysis in this paper, acknowledging that there may be important differences between jurisdictions in how these questions are answered.

We have argued that capable minor parents should be medical decision-makers for their children in the same way and to the same extent as capable adult parents. That is because the limits on decisions for minors about their own health are parallel to those that apply to all parental decision making. Capable minor parents should be permitted to consent to and refuse medical treatment within the zone of parental discretion. One significant advantage of this conclusion is that it simplifies assessment of cases like that of M. The same framework for decisions that applies to adult parents can be used for minor parents. Furthermore, the same approach to assessment of maturity and capacity can be applied as for decisions that minors might make for themselves.

None of the above arguments imply that minor parents can make harmful decisions for their children, nor should bad decisions be blithely accepted. Parents should be supported by health professionals to make good decisions for their children. In the case, M should be counselled, encouraged and supported to consent to an LP for her baby, as this would be best for B. However, her refusal, if it persists, should be respected.

## Footnotes

This is a hypothetical case (though based on a composite of cases in our experience)

We will use the term ‘minors’ to refer generically to young people who have not reached the legal age of adulthood. Some jurisdictions distinguish between older minors s (Eg above the age of 16 or 17) and younger minors. Since our focus is on ethics rather than law, and we do not believe that there is a clear ethical rationale to distinguish (say) between 15 and 16 year olds, we will refer generically to the wider category of ‘minors’.

Approximately 0.3% of births in the UK in 2017 were to mothers under the age of 20 https://www.ons.gov.uk/peoplepopulationandcommunity/birthsdeathsandmarriages/livebirths/datasets/birthsbyparentscharacteristics

The focus of this paper will be for jurisdictions that do not automatically grant minor-parents a right to decide; we will note, for reference, the legal framework that applies to decisions in England and Wales. Coughlin, discusses minors further in: Medical decision-making in paediatrics: Infancy to adolescence, Kevin W Coughlin, Paediatr Child Health. 2018 May; 23(2): 138–146

The basis of parental rights is complex and a full discussion is beyond the scope of this paper. We will accept for the sake of this paper that parents should have discretion (within a range) over medical decisions for their children. Of course, for those who reject such a view, neither adult parents, nor minor-parents should be free to make medical decisions for their children. Ref Brake, Elizabeth and Millum, Joseph, "Parenthood and Procreation", The Stanford Encyclopedia of Philosophy (Spring 2018 Edition), Edward N. Zalta (ed.), URL = <https://plato.stanford.edu/archives/spr2018/entries/parenthood/

In England and Wales, he would acquire legal parental authority if he were married to M at the time of birth, or registered B with M or gained a parental responsibility agreement from M or a parental agreement order from the courts.

In England and Wales, B’s grandmother would only have a legal right to make decisions for B if she were granted parental responsibility by a court order. Children act 1989, part 1, section 5

One limit to parental discretion over decisions is the finite nature of medical resources. For example, in the case, the cost of a prolonged hospital stay might provide one reason not to allow parents to refuse a lumbar puncture. This consideration, however, would apply equally to decisions made by adult or minor parents. Harm isn’t all you need: parental discretion and medical decisions for a child, Wilkinson D, Nair T, J Med Ethics, 2016 Feb;42(2):116-8

For example, in England and Wales, Jehovah’s Witness teenagers have usually been overruled when they have refused blood transfusions. Re P (Medical Treatment: Best Interests) [2003] EWHC 2327 (Fam); [2004] 2 F.L.R. 1117; [2004] Fam. Law 716; Re E (A Minor) (Wardship: Medical Treatment) [1993] 1 F.L.R. 386; [1994] 5 Med. L.R. 73. In a recent, case, a judge overruled a teenager who was refusing Insulin, commenting that “the law is clear that the court is not mandated to accept the wishes and feelings of a competent child where to honour those wishes and feelings would result in manifest, and even fatal, harm to that child.” University Hospitals Plymouth NHS Trust v B (Urgent Medical Treatment) [2019] EWHC 1670 (Fam)

Although we have argued and accepted that mature minors should have restricted autonomy to make medical decisions, our argument here would hold *even if* someone were of the view that minors with capacity should be permitted to refuse treatment. On that view, a mature-minor should have the same rights as an adult to make decisions – and therefore the zone of parental discretion would apply to decisions for their children.

RETRACTED: Ileal-lymphoid-nodular hyperplasia, non-specific colitis, and pervasive developmental disorder in children, Dr AJ Wakefield, SH Murch, A Anthony et al, Lancet Volume 351, P637-641, February 28, 1998
